# The Antimicrobial Peptide MPX Can Kill *Staphylococcus aureus*, Reduce Biofilm Formation, and Effectively Treat Bacterial Skin Infections in Mice

**DOI:** 10.3389/fvets.2022.819921

**Published:** 2022-03-29

**Authors:** Chunling Zhu, Yaya Zhao, Xueqin Zhao, Shanqin Liu, Xiaojing Xia, Shouping Zhang, Yimin Wang, Huihui Zhang, Yanzhao Xu, Shijun Chen, Jinqing Jiang, Yundi Wu, Xilong Wu, Gaiping Zhang, Yueyu Bai, Jianhe Hu, Hanna Fotina, Lei Wang, Xueming Zhang

**Affiliations:** ^1^College of Animal Science and Veterinary Medicine, Henan Institute of Science and Technology, Xinxiang, China; ^2^College of Veterinary Medicine, Jilin University, Changchun, China; ^3^Faculty of Veterinary Medicine, Sumy National Agrarian University, Sumy, Ukraine; ^4^School of Chemistry and Chemical Engineering, Henan Institute of Science and Technology, Xinxiang, China; ^5^State Key Laboratory of Marine Resource Utilization in South China Sea, School of Biomedical Engineering, Hainan University, Haikou, China

**Keywords:** antimicrobial peptide MPX, *Staphylococcus aureus*, membrane destruction, wound healing, inflammation

## Abstract

*Staphylococcus aureus* is a common pathogen that can cause pneumonia and a variety of skin diseases. Skin injuries have a high risk of colonization by *S. aureus*, which increases morbidity and mortality. Due to the emergence of multidrug-resistant strains, antimicrobial peptides are considered to be among the best alternatives to antibiotics due to their unique mechanism of action and other characteristics. MPX is an antibacterial peptide extracted from wasp venom that has antibacterial activity against a variety of bacteria. This study revealed that MPX has good bactericidal activity against *S. aureus* and that its minimum inhibitory concentration (MIC) is 0.08 μM. MPX (4×MIC) can kill 99.9% of bacteria within 1 h, and MPX has good stability. The research on the bactericidal mechanism found that MPX could destroy the membrane integrity, increase the membrane permeability, change the membrane electromotive force, and cause cellular content leakage, resulting in bactericidal activity. Results from a mouse scratch model experiment results show that MPX can inhibit colonization by *S. aureus*, which reduces the wound size, decreases inflammation, and promotes wound healing. This study reports the activity of MPX against *S. aureus* and its mechanism and reveals the ability of MPX to treat *S. aureus* infection in mice, laying the foundation for the development of new drugs for bacterial infections.

## Introduction

*Staphylococcus aureus* is a very common Gram-positive pathogen that occurs widely in the natural environment (1). *S. aureus* can cause a variety of infectious diseases, such as skin and soft tissue infections, pneumonia and bacteremia (2). *S. aureus* is a common cause of human skin infections, especially in damaged skin, where there is an increased risk of *S. aureus* colonization and infection (3). *S. aureus* infections were previously treated with antibiotics such as penicillin, aminopenicillin, tetracycline, and cephalosporin. However, in recent decades, the number of *S. aureus* infections has increased dramatically, accompanied by the emergence of resistant strains. In particular, the emergence of multidrug-resistant (MDR) strains poses a serious threat to public health (4). A study in Brazil showed that in 536 swabs collected from patients in a teaching hospital and the ICU environment, the detection rate of methicillin-resistant *S. aureus* was 20.6%, and multi-drug-resistant strains spread between patients and pathogens (5). At present, only a few compounds, such as vancomycin, a “last-resort drug”, can still effectively treat *S. aureus* infections. Vancomycin is an antibiotic widely used in hospital environments, especially against methicillin-resistant Staphylococcus. The prevalence of methicillin-resistant *S. aureus* has led to a significant increase in vancomycin use which has accelerated the development of vancomycin-resistant *S. aureus*. Currently, the emergence of vancomycin-resistant *S. aureus* strains (VRSA) strains has become a global problem, and it is urgent to find effective alternatives to antibiotics (4, 6).

Antimicrobial peptides (AMPs), also known as host defense peptides (HDPs), are an important part of the innate immune system (7). AMPs have a wide range of bactericidal effects, and broad-target short cationic compounds have a membrane-decomposing effect on negatively charged microbial membranes. Therefore, they can inhibit the proliferation of a variety of bacteria, viruses, fungi and protozoa (8). The antibacterial mechanism of AMPs is different from that of existing antibiotics. Studies have shown that AMPs prevent bacterial metabolism by restructuring the distribution of liposome domains, leading to bacterial death (9). In recent years, the synthesis and modification of AMPs has also become a hot research topic. AMPs destroy bacteria by physically destroying the structure of cell membranes, making it difficult for bacteria to develop drug resistance (10). Therefore, antimicrobial peptides are expected to become one of the best alternatives to antibiotics.

Mastoparan (MP) peptides are isolated from the venom of Vespidae insects that use it to immobilize their prey (11). MPX is a 14-amino-acid peptide with a net positive charge of +4 that belongs to the MP family of AMPs, and is found in high concentrations in wasp venom (12). Early experiments in our laboratory revealed that MPX can effectively protect mice against the Gram-negative bacterium *Actinobacillus pleuropneumoniae* against lung damage, providing a basis for research on the treatment of respiratory infections (13). This study explored the antibacterial effect of MPX against the Gram-positive bacterium *S. aureus*. In this study, we found that MPX has good bactericidal activity against *S. aureus* and can exert a bactericidal effect by inhibiting biofilm formation, destroying bacterial membrane integrity, and inducing cellular content release. MPX also has a certain degree of stability. In addition, the ability of MPX to treat *S. aureus* wound infection *in vivo* was further evaluated in a mouse scratch model.

## Materials and Methods

### Animals

Thirty BALB/c mice (6–8 weeks old, weight 18–20 g, female mice) were purchased from Zhengzhou University Animal Center (No. 41003100024648). All animal studies were conducted in accordance with the experimental practices and standards of the Animal Welfare and Research Ethics Committee of Henan Institute of Science and Technology.

### Reagents

A BCA protein kit was purchased from Biyuntian Technology. PBS, LB medium, crystal violet, NaCl, KCl, CaCl_2_, MgCl_2_, 96-well culture plates, N-phenyl-1-naphthylamine(NPN), and 3,3'-dipropylthiadicarbocyanine iodide(DiSC3-5) were purchased from Zhibao Biotechnology Co., Ltd.

### Peptide Synthesis

The MPX (H-INWKGIAAMAKKLL-NH_2_; Mw: 1556.01) was produced from Shanghai Jier Biochemical Company (China) using a solid-phase N-9-fluoromethoxycarbonyl (Fmoc) strategy and high-performance liquid chromatography (HPLC) purification, and its purity was as high as 98%.

### Antibacterial Activity

The agar diffusion test was used to detect the antibacterial effect of MPX on *S. aureus* ATCC 25923. *S. aureus* ATCC 25923 was cultured overnight, and the supernatant was discarded by centrifugation. The bacteria were washed with sterile PBS, and resuspended in a smaller volume to concentrate the cells. Next, 50 μL of the bacterial solution was pipetted into 15 mL of agar and shaken gently to mix. The plate was poured, a puncher was used to make holes after the agar medium had solidified and 20 μL of 1 mg/mL MPX was added to the wells. Sterile water was used as a control. The plates were incubated overnight at 37°C and the results were observed.

To determine the minimum inhibitory concentration (MIC) (14), *S. aureus* ATCC 25923 was cultured overnight and adjusted to 10^5^ CFU/mL. Next, 100 μL of the adjusted bacterial solution was added to each well of a 96-well polypropylene microtiter plate. After serially two-fold dilution of the drug, 100 μL was added to a 96-well plate to obtain concentrations from 0.0025 to 0.32 μM. The solutions in the wells were mixed, and controls were established at the same time: a positive control with only the bacterial solution without AMPs and a blank control with only the culture medium. The plates were incubated overnight at 37°C to observe the antibacterial activity results.

### Killing Curve

*S. aureus* was cultivated, the OD_600_ of the bacterial solution was adjusted to approximately 1.0, and MPX (1×MIC, 4×MIC) was added. In addition, sterile water was used as a control. The samples were incubated with shaking at 37°C. The OD_600_ value of the bacterial solution was measured every 1 h, and plate counting was performed at the same time to obtain the antibacterial time-killing curve of MPX against *S. aureus* ATCC 25923 (15).

### Stability

To detect the influence of salt ions, pH, temperature, and repeated freezing and thawing on the antibacterial activity of MPX, MPX was incubated with different concentration NaCl, KCl, CaCl_2_, and MgCl_2_ solutions (0, 50, 100, 150, and 200 mmol/mL) for 30 min. Solutions with different pH values were prepared with HCl and NaOH, producing pH values of 4, 5, 6, 7, 8, 9, 10, and MPX was incubated in these solutions for 30 min. MPX solutions were also incubated at different temperatures for 30 min (0, 25, 50, 75, and 100°C). A 1 mg/mL MPX solution was repeatedly frozen and thawed 0, 2, 4, 6, 8, 10, and 12 times. Ten microlitres of each of the peptide solutions treated as described above was collected, and the changes in the antimicrobial activity of the AMPs were detected by the agar diffusion assay.

An MPX protease stability assay was performed in the following manner. MPX (1.3 μM) was mixed with equal volumes of proteinase K and trypsin at different concentrations. After incubating at 37°C for 30 min, the samples were incubated in a boiling water bath for 5 min to stop the protease reaction. The protease stability of MPX was detected by the agar diffusion assay.

### BCA Protein Assay

*S. aureus* was cultured overnight, washed and resuspended in sterile PBS. MPX (1×MIC, 4×MIC) was added to the bacterial solution. Sterile water was added as a control. A peptide-only group was a negative control. The mixture was allowed to stand for 5 min, and the supernatant was collected after centrifugation. The effect of MPX on the permeability of *S. aureus* ATCC 25923 for protein was analyzed according to the instructions of the BCA kit.

### Propidium Iodide Uptake

*S. aureus* was centrifuged, washed, and resuspended in sterile PBS. MPX (4×MIC) was added to the bacterial solution. The bacteria were cultured with shaking at 37°C and then centrifuged and resuspended in PBS solution. The cells were incubated with propidium iodide (PI) stain for 10 min in the dark. The suspension was fixed on a clean glass slide and observed with a fluorescence microscope.

### Crystal Violet Assay

An overnight culture of *S. aureus* was inoculated into a 96-well polystyrene microtiter plate at a ratio of 1%. MPX (0.25×MIC, 0.5×MIC and 1×MIC) was added to the wells. Sterile water was used as a control. The plates were incubated at 37°C for 24 h. The microplates were then washed 3 times with sterile PBS, and the cells were fixed with 70% methanol for 30 min. Then, the cells were stained with 1% Hucker crystal violet staining solution at room temperature for 5 min. The dyeing solution was aspirated, and the cells were rinsed with water until no color remained. Images were acquired after drying. Then, 100 μL of a 70% ethanol solution was added to each well to dissolve the residual dye, and a microplate reader was used to measure the absorbance at OD_570_ nm (16).

### Observation of Biofilm Formation by Confocal Laser Microscopy

*S. aureus* ATCC 25923 was cultivated as described above. After culturing in a 37°C incubator for 24 h, MPX (final concentration: 4×MIC) was added to the sample. The control did not have any added peptide. *S. aureus* was washed 3 times with 0.85% NaCl to remove nucleic acids and other medium components. A LIVE/DEAD BacLight Bacterial Viability L-7012 Kit (Molecular Probes, Eugene, OR, USA), containing two component dyes (SYTO 9 and PI in a 1:1 mixture) in solution, was used prior to microscopy and quantitative assays according to the test instructions. A total of 3 μL of the dye mixture was added to each well, the wells were incubated at room temperature in the dark for 15 min, and the bacterial survival in the biofilm was observed by confocal laser microscopy.

### NPN Uptake

*S. aureus* ATCC 25923 was cultured overnight in a 96-well plate. An NPN solution was added to a final concentration of 10 μM. The solutions were mixed well and incubated for 20 min. MPX (1×MIC, 4×MIC) was added to the wells. Sterile water was used as the control, and ampicillin (Amp) (0.134 μM/50 μg/mL) was used as the positive control. The excitation wavelength was 350 nm, the emission wavelength was 420 nm, and the fluorescence intensity was detected by a microplate reader (13).

### DiSC3-5 Assay for Detection of Membrane Potential

Overnight-cultured *S. aureus* ATCC 25923 was centrifuged and resuspended in sterile PBS. A DiSC3-5 solution was added to a final concentration of 1 μM. The cells were incubated in the dark for 20 min. MPX (1×MIC, 2×MIC and 4×MIC) was added. Sterile water was used as the control, and AMP (0.134 μM/50 μg/mL) was used as the positive control. The excitation wavelength was 622 nm, the emission wavelength was 670 nm, and the fluorescence intensity was detected by a microplate reader.

### Scanning Electron Microscopy

A sterile slides were placed in 6-well cell culture plates and bacteria were allowed to grow naturally on the slides to form a biofilm. Overnight culture broth and fresh broth were added at a volume ratio of 1:100, and incubated at 37°C for 24 h. Different final concentrations of MPX (1×MIC, 4×MIC) were added, incubation was continued. Then, a 2.5% glutaraldehyde solution was added to each well, and the cells were fixed at room temperature for 30 min. The plate was rinsed 3 times with PBS for 10 min each time. Gradient dehydration was performed with increasing concentrations of an ethanol solution (30, 50, 70, 80, 90, 95, and 100%). After ion spray gold coating, biofilm formation was observed by scanning electron microscopy (SEM) (17).

### Transmission Electron Microscopy

Transmission electron microscopy (TEM) was used to detect the bacterial structure changes after MPX acted on *S. aureus* ATCC 25923. *S. aureus* was cultured overnight. MPX was added to different final concentrations. The cells were incubated with shaking at 37°C for 2 h, and then sodium phosphotungstate was used for negative staining. The effect of AMPs on *S. aureus* was observed. Centrifugation, fixation, dehydration, soaking, embedding and polymerization, sectioning and staining of the culture were performed. Finally, a transmission electron microscope was used to observe the results.

### Ointment Preparation

Stearyl alcohol, glyceryl monostearate and white petrolatum were melted in a water bath at 80°C. MPX was mixed into glycerine and sodium lauryl sulfate predissolved in distilled water and heated to 80°C. Then, the above combination was mixed well and stirred until it condensed. Thus, an antibacterial peptide ointment was obtained.

### Mouse Skin Injury Model

Taking female BALB/c mice as the research object, a mouse skin injury model was established. The mice were divided into three groups. The mice were anesthetized with ether, and the back of the mice was shaved and then depilated with a depilatory cream. The back was wiped with an alcohol-soakedcotton ball. The back skins of the first two groups of mice were injured with medical tape. *S. aureus* ATCC 25923 was cultured overnight in log phase. A total of 2.5×10^9^ CFU was added dropwise to the wound of each mouse for infection. One day after bacterial infection, MPX ointment was applied for treatment in the second group. After that, the treatment was applied each day in the morning and evening. The third group was the untreated blank control group. The wounded area of the skin was measured and photographed every day.

### Colonization

Six days after the mice were infected with *S. aureus* ATCC 25923, the skin around the wounds of the mice was gently wiped with alcohol-soaked cotton, and the partial skins of the backs of the mice were excised and weighed. Then, the skin was ground and plated.

### Histology

After *S. aureus* infected the mice, the skin and organs of the mice were removed on the second day and the sixth day after infection. Then, 4% paraformaldehyde fixation, paraffin embedding, sectioning, HE staining, and electron microscope observation of pathological changes were performed. For immunohistochemistry, the skin samples were fixed in 4% paraformaldehyde, embedded, and sectioned, and then the sections were processed. The sections were incubated with an anti-Ly6g antibody to observe the infiltration of neutrophils. For examination of the cellular ultrastructure, mouse wound skin was excised and fixed with a glutaraldehyde solution. After the tissue sections were prepared, they were observed by TEM.

### Statistical Analysis

GraphPad Prism software (version 8.0, La Jolla, CA, USA) was used to perform statistical analyses. All results are expressed as the mean ± standard deviation. One- way analysis of variance (ANOVA) followed by Tukey's test was used to perform group comparisons. Statistical significance is expressed as follows: ^*^P < 0.05; ^**^P < 0.01; ^***^P < 0.001.

## Results

### The Antimicrobial Peptide MPX Shows Bactericidal Activity Against *S. aureus*

**Figure 1:**
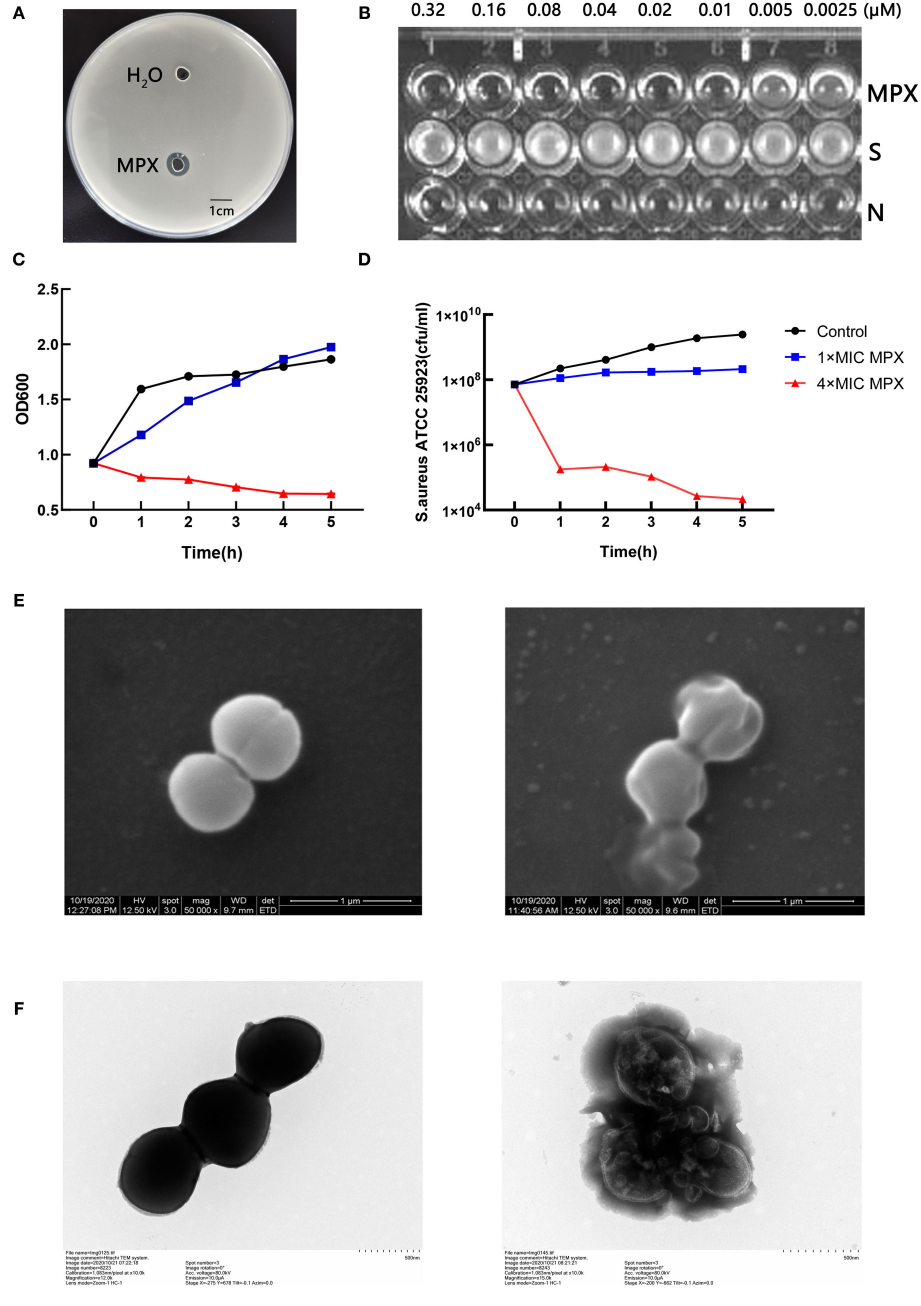
Detection of the bactericidal activity of the antimicrobial peptide MPX. **(A)** The agar diffusion test of the antimicrobial activity of MPX against *S. aureus* ATCC 25923. **(B)** MIC results. S: *S. aureus* ATCC 25923; N: blank LB medium. **(C)** OD_600_ measurement of bacterial culture after the action of MPX for different times. **(D)** The determination of the number of viable bacteria after the action of MPX for different times. **(E)** Scanning electron microscopy observation of the effect of MPX on *S. aureus* ATCC 25923. The left picture shows the normal morphology of *S. aureus*, and the right picture shows the bacterial morphology of *S. aureus* after MPX (1×MIC) exposure. **(F)** Transmission electron microscopy observation of the effect of MPX on *S. aureus* ATCC 25923. The left picture shows the normal morphology of *S. aureus*, and the right picture shows the bacterial morphology of *S. aureus* after MPX (1×MIC) exposure. Error bars indicate the mean ± SEM, *n* = 3. Statistical significance was defined as **p < 0.01 and ***p < 0.001.

The antibacterial activity of MPX against *S. aureus* ATCC 25923 was detected by the agar diffusion method. The results are shown in Figure 1A. It was found that 1 mg/mL MPX produced a zone of inhibition against *S. aureus* ATCC 25923, while the control had no zone of inhibition, indicating that the antimicrobial peptide MPX has antibacterial activity against *S. aureus*. Furthermore, the MIC results in Figure 1B show that the MIC of MPX against *S. aureus* ATCC 25923 is 0.02 μM. The killing curve results are shown in **Figures 1C,D**. MPX (1×MIC) had relatively stable inhibition of *S. aureus* within 5 h. MPX (4×MIC) killed 99.9% of *S.aureus* ATCC 25923 within 1 h and produced good bactericidal activity. The electron microscopy results showed (Figures 1E,F) that MPX (1×MIC) caused *S. aureus* to shrink and leak cell contents, indicating antibacterial activity against *S. aureus*, while in the blank control, the structure of *S. aureus* was intact. The above experimental results show that MPX has good bactericidal activity against *S. aureus* ATCC 25923.

### MPX Is a Stable Antimicrobial Peptide

**Figure 2:**
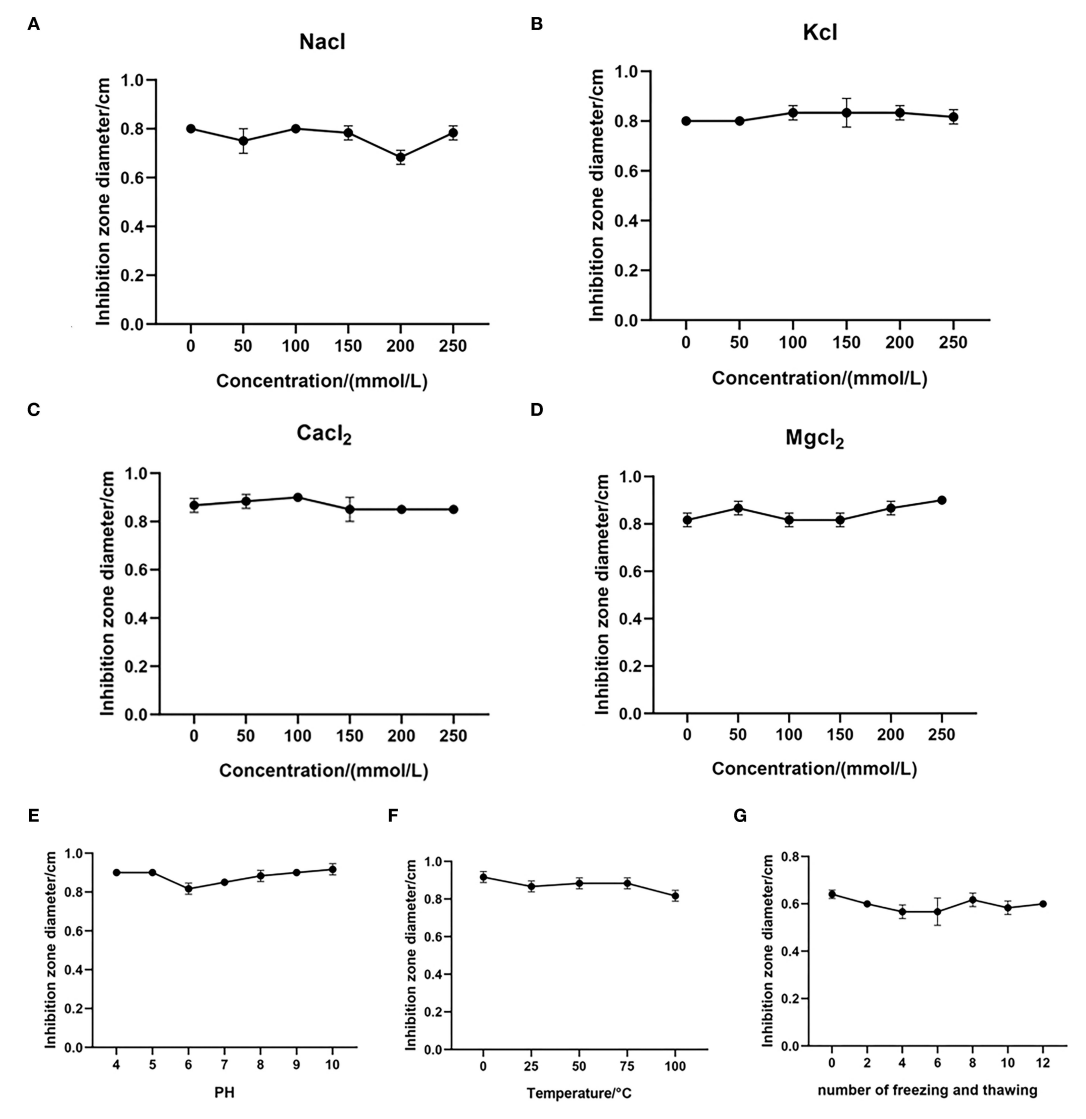
Stability test of MPX. **(A–D)** The effect of different concentrations of NaCl, KCl, CaCl_2_ and MgCl_2_ on the antibacterial activity of MPX. **(E)** The effect of different temperatures on the antibacterial activity of MPX. **(F)** The effect of different pH values on the antibacterial activity of MPX. **(G)** The effect of repeated freezing and thawing on the antibacterial activity of MPX. Error bars indicate the mean ± SEM, *n* = 3.

To study the effect of the monovalent cations Na^+^ and K^+^ on the antibacterial activity of MPX, the antibacterial agar diffusion method was used to determine the MIC of MPX in different concentrations of NaCl and KCl (50, 100, 150, 200, and 250 mmol/mL) and the effect of the ions on the antibacterial activity against *S. aureus* after incubation. As shown in Figures 2A,B, NaCl and KCl had little effect on the antibacterial activity of MPX as their concentrations increased. This result shows that MPX has good stability in the presence of Na^+^ and K^+^.

At the same time, the effects of the divalent cations Ca^2+^ and Mg^2+^ on the antibacterial activity of MPX were tested. The effect on MPX antibacterial activity against *S. aureus* after incubation with different concentration CaCl_2_ and MgCl_2_ solutions (50, 100, 150, 200, and 250 mmol/mL) was determined. As shown in Figures 2C,D, CaCl_2_ and MgCl_2_ had no significant effect on the antibacterial activity of MPX as their concentration increased. This solutions shows that the antibacterial activity of MPX against *S. aureus* has good stability in the presence Ca^2+^ and Mg^2+^.

Furthermore, the antibacterial activity of MPX against *S. aureus* was determined at different temperatures and pH values. As shown in Figure 2E, MPX was subjected to different temperature treatments, and the maximum temperature reached 100°C. Temperature had basically no effect on the antibacterial activity of MPX, indicating that MPX has good thermal stability. As shown in Figure 2F, pH in the range of 4–10 had basically no effect on MPX activity, indicating that MPX activity is relatively stable in acidic and weakly alkaline environments.

The antibacterial activity of MPX after repeated freezing and thawing (0, 2, 4, 6, 8, 10, and 12 times) was determined. The results are shown in Figure 2G. After repeated freezing and thawing 0–12 times, the antimicrobial activity of MPX did not change significantly, indicating that MPX has good stability against repeated freezing and thawing.

Furthermore, proteolytic instability is an enormous obstacle for AMPs to overcome in order to function as antibiotics. As shown in Supplementary Figures 1, 2, after the action of proteinase K and trypsin, the antibacterial activity of MPX against *S. aureus* ATCC 25923 was decreased. Proteinase K at 40 μg/mL significantly reduced the antibacterial activity of MPX. When 100 μg/mL proteinase K acted on MPX, the residual antibacterial activity decreased to 66.3%. The experimental results show that MPX has certain components resistant to proteinase K hydrolysis. 20 μg/mL of trypsin can significantly reduce the antibacterial activity of MPX. When 100 μg/mL trypsin acted on MPX, the residual antibacterial activity decreased to 19.6%. The experimental results show that MPX has poor tolerance to trypsin.

### MPX Destroys the Integrity of the Bacterial Cell Membrane, Changes the Membrane Potential, and Releases Cell Contents

**Figure 3:**
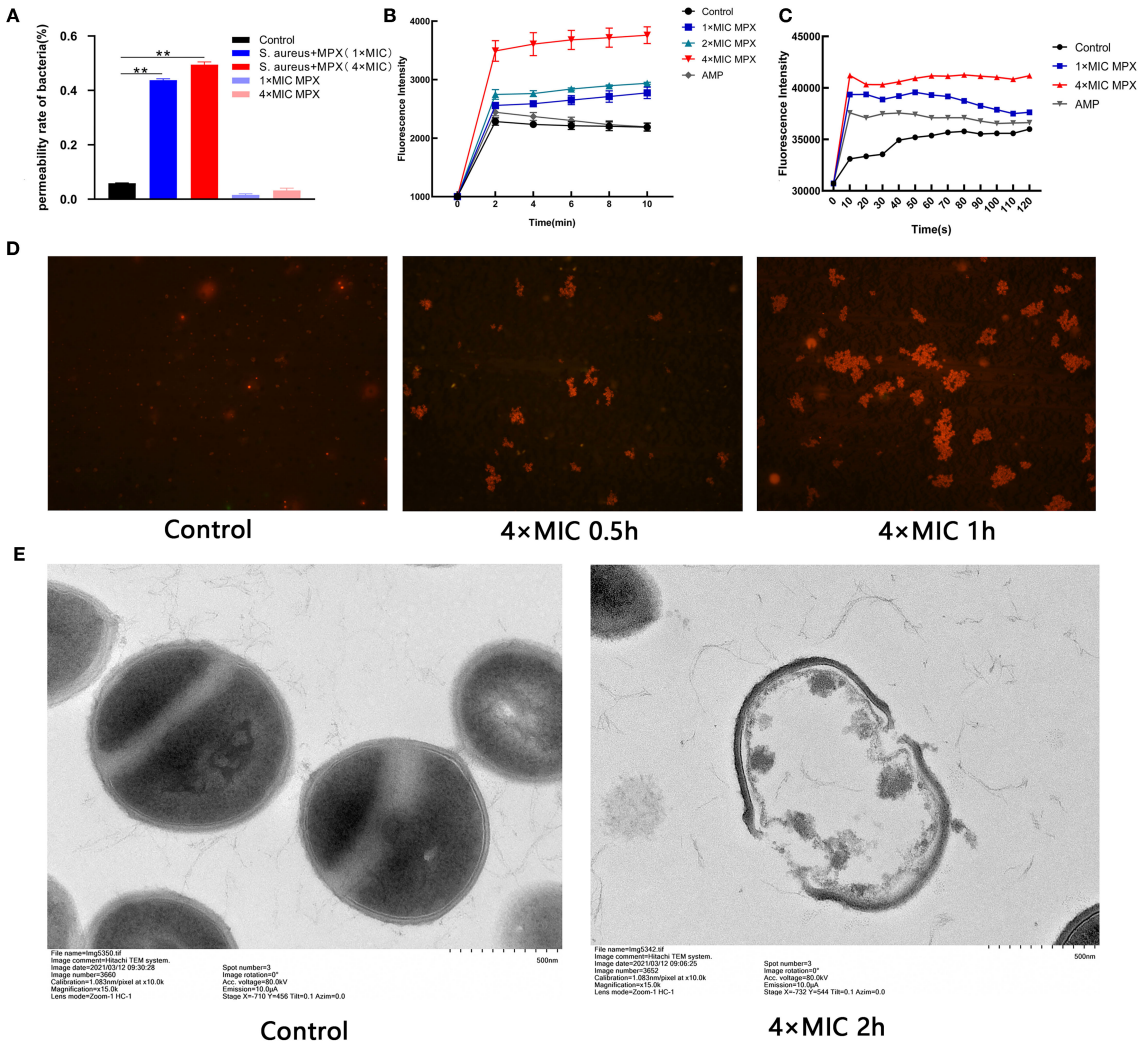
The mechanism of action of MPX activity on *S. aureus*. **(A)** The BCA kit was used to detect the results of total protein content in bacterial supernatant. **(B)** Uptake of NPN by *S. aureus* treated with MPX. **(C)** DiSC3-5 was used to detect changes in the membrane potential of *S. aureus* after MPX treatment. **(D)** Fluorescence microscopy observation of PI staining after MPX treatment. **(E)** Visual observation of the mechanism of MPX on *S. aureus* ATCC 25923 by transmission electron microscopy. Error bars indicate the mean ± SEM, *n* = 3. Statistical significance was defined as **p < 0.01.

In the previous experiments, it was shown that MPX has killing activity against *S. aureus*. To further explore the *S. aureus* killing mechanism of MPX, the effect of MPX on the cell membrane structure of *S. aureus* was studied. A BCA protein quantitation kit was used to determine the changes in the total protein content of the bacterial supernatant after the action of MPX. The results are shown in Figure 3A. The results showed that the total protein content of the bacterial culture supernatant was significantly higher than that of the control group after 5 min of MPX acting on *S. aureus* (*P* < 0.01).

To detect whether MPX destroys the integrity of the bacterial membrane after it acts on *S. aureus*, the NPN probe was used to evaluate the membrane destruction mechanism of MPX. NPN is a hydrophobic fluorescent reagent that can emit strong fluorescence in a hydrophobic environment. When the cell membrane is destroyed, NPN enters the hydrophobic environment and releases strong fluorescence. As shown in Figure 3B, MPX (1×MIC, 2×MIC, 4×MIC) acted on *S. aureus* for 0, 2, 4, 6, 8, and 10 min, and then the NPN fluorescence intensity was measured. Our result revealed that MPX rapidly destroyed the bacterial membrane of *S. aureus* in a concentration-dependent manner. Even at a concentration of 0.02 μM (1×MIC), it destroyed the integrity of the *S. aureus* membrane, and the fluorescence intensity was significantly higher than that of the control group and the antibiotic AMP treatment group.

DiSC3-5 is a cationic fluorescent probe that is sensitive to cell membrane potential. When the cell is in a polarized state, DiSC3-5 exhibits self-quenching of fluorescence. When the cell membrane is depolarized, DiSC3-5 emits detectable fluorescence. The results are shown in Figure 3C. It was found that the antimicrobial peptide MPX rapidly destroyed the polarization state of the *S. aureus* cell membrane in a concentration-dependent manner. After MPX (1×MIC, 4×MIC) acted on *S. aureus*, the bacteria were depolarized within 10 s. The intensity of fluorescence produced by MPX (1×MIC) was higher than that produced by the antibiotic AMP.

PI cannot stain live cells. However, when the bacterial cell membrane is destroyed, PI can stain the nucleus after passing through the cell membrane. The results are shown in Figure 3D. After MPX (4×MIC) acted on *S. aureus* for 0.5 h, the number of PI-stained bacteria was significantly increased compared with that in the treatment group without peptide. Especially after 1 h of action, the MPX severely destroyed the bacterial cell membrane, PI entered the bacteria in large quantities, and the number of dead bacteria increased significantly.

To further visually evaluate the mechanism of action of MPX on *S. aureus*, TEM was used to observe the morphological changes of *S. aureus* after exposure to MPX. The result is shown in Figure 3E. The surface of *S. aureus* without peptide action was smooth and bright, and the structure was intact. After the action of MPX (4×MIC), the cell membrane of *S. aureus* was severely damaged, the cell contents leaked out, and the structure was disrupted. The above experimental results show that MPX can increase the permeability of PI, change the integrity of the cell membrane, and promote the leakage of cellular contents, thereby producing a bactericidal effect.

### MPX Reduces the Formation of Bacterial Biofilms

**Figure 4:**
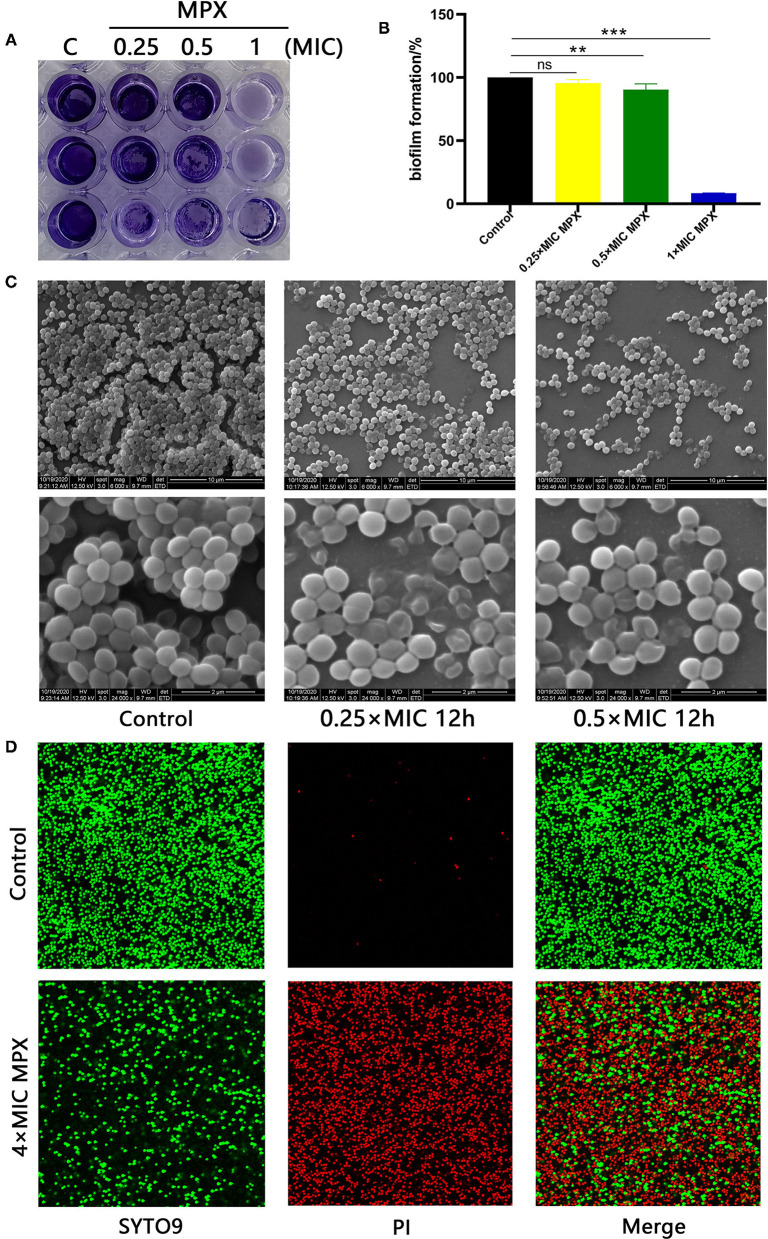
MPX inhibits biofilm formation. **(A)** The results of crystal violet staining of biofilms after different concentrations of MPX were applied to *S. aureus* ATCC 25923. **(B)** OD_570_ measurement after crystal violet was dissolved in 70% ethanol. **(C)** After exposure of *S. aureus* ATCC 25923 to different concentrations of MPX, scanning electron microscopy was used to observe the biofilm. **(D)** The results of staining *S. aureus* with SYTO 9/propidium iodide. Error bars indicate the mean ± SEM, *n* = 3. Statistical significance was defined as follows: **p < 0.01; ***P < 0.001; ns *P* > 0.05.

Bacterial biofilms are of great significance to the adaptive growth of bacteria and the development of resistance to bactericidal drugs. To study the effect of MPX on *S. aureus* biofilms, different concentrations of MPX (0.25×MIC, 0.5×MIC, 1×MIC) were incubated with *S. aureus* for 24 h. Observation by crystal violet staining revealed that MPX can inhibit the formation of *S. aureus* biofilms (Figure 4A). After 70% ethanol was used to dissolve crystal violet, the OD_570_ measurement results showed that an MPX (0.5×MIC) significantly reduced *S. aureus* biofilm formation (^**^: *p* < 0.01, Figure 4B).

The effect of MPX on *S. aureus* biofilm formation was further observed by SEM. MPX (1×MIC, 4×MIC) was incubated with *S. aureus* for 24 h. The results are shown in Figure 4C. In the blank control, the biofilm formed by *S. aureus* was dense, and the gaps between bacterial were small. After the action of MPX (1×MIC), *S. aureus* biofilm formation was significantly reduced, and the structure was loose. This result was consistent with the crystal violet staining results. After the action of MPX (4×MIC), *S. aureus* biofilm formation was further reduced, and the bacteria were more dispersed. The confocal laser microscopy results showed that the number of dead bacteria in the *S. aureus* biofilm significantly increased and that the number of viable bacteria decreased significantly with the addition of MPX (Figure 4D). The above experiments show that MPX can inhibit S. *aureus* biofilm formation.

### MPX Ointment Promotes Wound Healing and Reduces Bacterial Colonization

**Figure 5:**
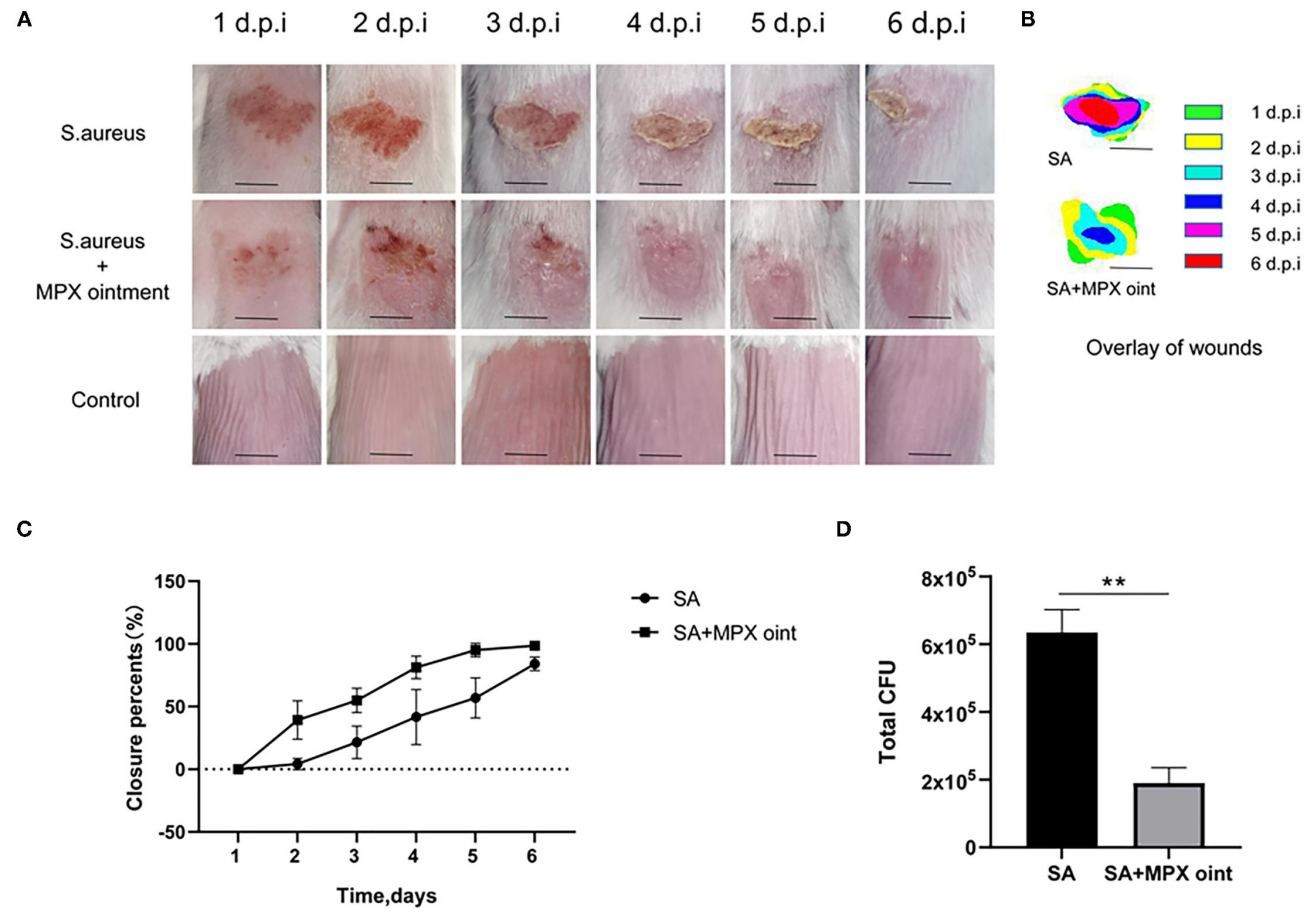
MPX ointment can promote skin wound healing in mice infected with *S. aureus* ATCC 25923. Mice in each group were infected with *S. aureus* ATCC 25923 without treatment (*S. aureus*/SA) or with treatment with MPX ointment (*S. aureus*+MPX ointment/SA+MPX oint), and mice in an uninfected group did not receive any treatment (Control). **(A,B)** Wound pictures and superimposed pictures of mice infected with *S. aureus* 1, 2, 3, 4, 5, and 6 days later. The black line represents 0.5 cm; d.p.i: days post infection. **(C)** Wound healing rate. **(D)** The number of colonies at the wound site after the sixth day of *S. aureus* infection. Error bars indicate the mean ± SEM, *n* = 3. Statistical significance was defined as **p < 0.01.

We established a mouse skin scratch model to evaluate the wound healing activity of MPX. The results are shown in Figures 5A,B. Mice were inoculated with *S. aureus* on the scratched back, and the wounds of the mice in the untreated group did not heal completely by the sixth day after infection. The mice in the group treated with MPX ointment had significantly faster healing after infection. As shown in Figure 5C, the wounds in the treatment group were healed by 81.2% on the fourth day and more than 95% on the fifth and sixth days. In the untreated group, the wounds healed by only 41.7% on the fourth day, 56.9% on the fifth day, and 84.1% on the sixth day. On the sixth day after *S. aureus* infection, colony counting was performed on the wound site to understand the impact of MPX on colonization. As shown in Figure 5D, MPX significantly reduced *S. aureus* colonization. The above results indicate that MPX can reduce *S. aureus* colonization and promote wound healing.

### MPX Ointment Promotes Skin Repair and Reduces Inflammation

**Figure 6:**
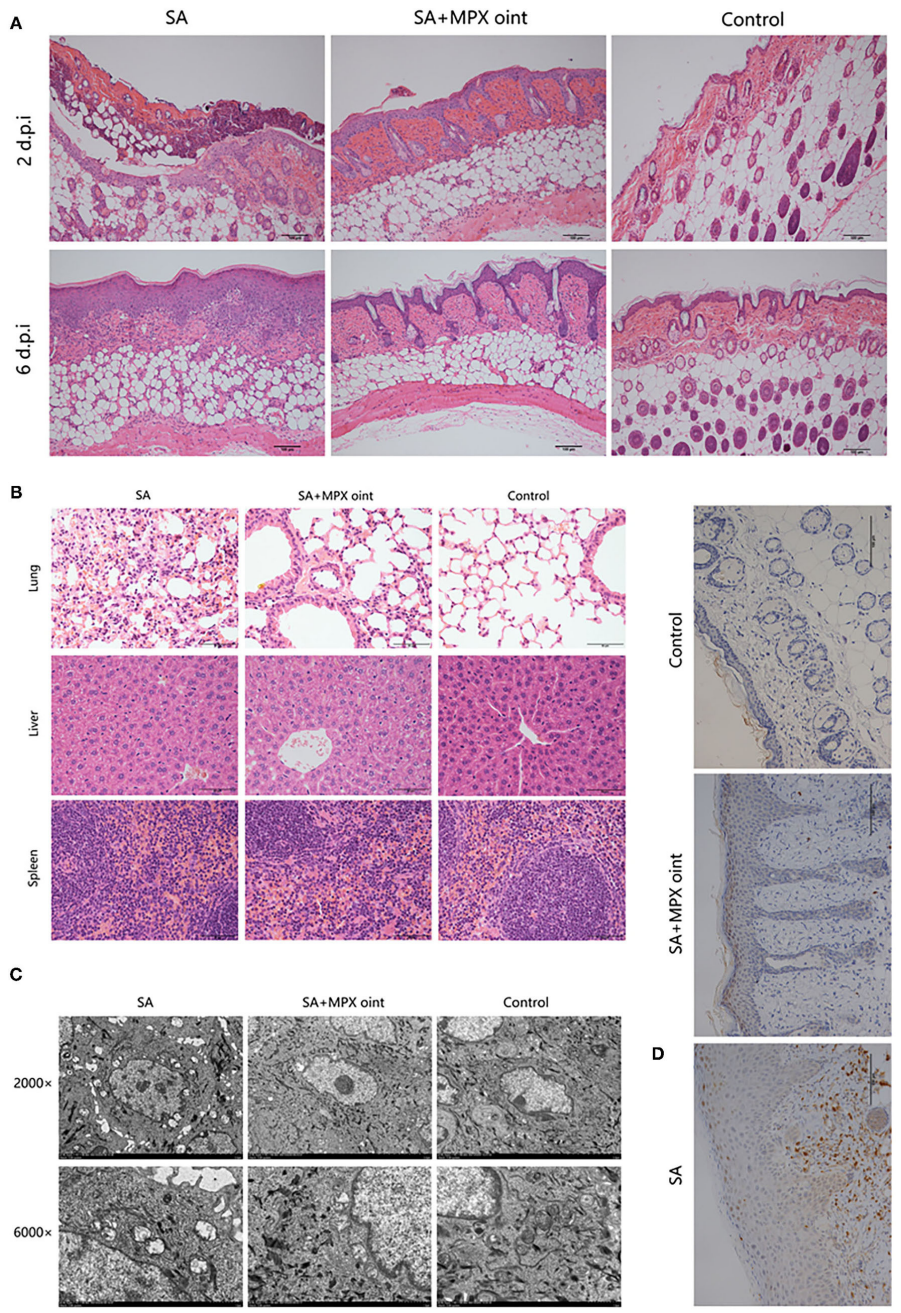
MPX ointment can promote skin repair and reduce inflammation. Mice in each group were infected with *S. aureus* ATCC 25923 without treatment (*S. aureus*/SA) or with treatment with MPX ointment (*S. aureus*+MPX ointment/SA+MPX oint), and mice in an uninfected group did not receive any treatment (Control). **(A)** The results of HE staining of the wounded skin on the 2nd and 6th days after infection with *S. aureus* ATCC 25923. **(B)** HE staining results of the lung, liver, and spleen on the 6th day after *S. aureus* ATCC 25923 infection. **(C)** Transmission electron microscopy to observe the ultramicrocellular structure of the wounded skin on the 6th day after *S. aureus* ATCC 25923 infection. **(D)** Immunohistochemical observation of Ly6G expression in wound skin on day 6 after infection with *S. aureus* ATCC 25923.

To further evaluate the anti-*S. aureus* effect of MPX in skin wound infection, we analyzed the histopathology of mouse skin lesions. On the second day after inoculation with *S. aureus*, the skin of mice in the non-antimicrobial peptide ointment treatment group showed infiltration by inflammatory cells and necrosis of the epidermis and hair follicles. After treatment with MPX ointment, although hair follicle necrosis and inflammatory cell infiltration appeared on the skin, the symptoms were milder than those in the untreated group. The blank control exhibited skin that was clearly organized and structured. Six days after inoculation, the mice in the untreated infection group still had a large amount of inflammatory cell infiltration, the thickness of the epidermis increased, the hair follicles were completely necrotic, and the structure was disordered. In the MPX ointment treatment group, there was only slight inflammatory cell infiltration on the sixth day, the structure was clear, the epidermis was regenerated, and the hair follicle glands were developed (Figure 6A). Changes in the tissue were observed on the sixth day after infection. The H&E staining results are shown in Figure 6B. The structure of the skin of mice in the blank control group was clear and normal. In the mice without MPX ointment treatment, the alveolar septum was congested, and oedema, inflammatory cell infiltration, and alveolar rupture, atrophy or even disappearance occurred. The MPX ointment treatment group showed mild inflammation, but the structure was clear. There were no obvious lesions in the liver or spleen. TEM was used to observe the ultrastructure of wound tissue cells in mice six days after infection with *S. aureus*. The results are shown in Figure 6C. The mitochondria of the skin tissue of the mice in the blank control group were round or nearly round, without swelling, and with clear ridges, a clear double-layer membrane structure, and extensive chromatin. The tissue cells of the mice in the *S. aureus* infection group without ointment treatment showed inconspicuous nucleoli, concentrated nuclear chromatin, chromatin margins, and mitochondria that were ruptured, swollen, vacuolated, and had blurred or even missing ridges. In the MPX ointment treatment group, the cell morphology was regular, and only some mitochondria were swollen. The infiltration of neutrophils in the wound skin of mice was analyzed by immunohistochemistry. It was found that there was a large amount of neutrophil infiltration in the infected group that was not treated with MPX ointment (Figure 6D). The MPX ointment treatment group showed reduced neutrophil infiltration. This result indicates that MPX ointment can reduce the level of local skin inflammation in the wound. The above experimental results showed that the antimicrobial peptide MPX can promote wound tissue recovery and wound healing and reduce inflammation.

## Discussion

*S. aureus* can cause a wide range of infections, which can lead to organ failure and death in severe cases. It is one of the most common pathogens in communities and hospitals worldwide. The emergence and rapid spread of methicillin-resistant *S. aureus* strains have produced great challenges for clinical treatment. According to reports, patients with methicillin-resistant *S. aureus* infection have an increased clinical risk, especially an increased risk of mortality (18). Therefore, we must strengthen the prevention of MDR *S. aureus* infection, find effective alternatives to antibiotics, and identify effective treatments. According to reports, the new photosensitizer S-PS (19), silver nanoparticles (AgNPs) (20), antimicrobial peptide sublancin (21), cell-penetrating peptides (CPPs) and antibiotic combination therapy (22) have good bactericidal activity against MDR *S. aureus*. Among these new antibacterial agents, antibacterial peptides have the potential to replace traditional antibiotics for the treatment of infections caused by MDR strains due to their antibacterial activity against a variety of pathogens. This study revealed that MPX can cause changes in the membrane structure of *S. aureus*, resulting in the leakage of contents and producing good antibacterial activity. MPX (4×MIC) killed 99.9% of *S. aureus*. Furthermore, MPX had good stability and maintained good antimicrobial activity in partial salt ion solutions, as well as good acid-base stability, thermal stability and repeated freeze–thaw stability. Previous research results from our laboratory showed that MPX also has good antibacterial activity against various serotypes of *Actinobacillus pleuropneumoniae*, has a low MIC, and can effectively prevent the invasion of *Actinobacillus pleuropneumoniae* in the respiratory system of mice (13). In contrast to the results in this study, although MPX has good thermal stability, pH stability and monovalent salt ion stability, a previous experiment showed that Ca^2+^ and Mg^2+^ can significantly inhibit the killing effect of MPX on *A. pleuropneumoniae*. The difference in stability may be caused by the different mechanisms of action of MPX on Gram-positive and Gram-negative bacteria. From the above experiments, it can be concluded that MPX has the potential to become an effective substitute for traditional antibiotic therapy.

To date, although the mechanism of action of AMPs has not been fully elucidated, most researchers agree that the bactericidal mechanism of AMPs occurs mainly via introduction of holes on the surface of bacteria, destruction of the membrane structure, and induction of a large amount of bacterial cell content leakage, thereby mediating bacterial death. It has been reported that AMPs can increase the permeability of cell membranes, alter the integrity of cell membranes, and cause the release of ions (23–25). In addition, AMPs can enter cells and bind to DNA, mediate bacterial death, and inhibit biofilm formation (26). Previous research results from our laboratory showed that MPX can increase the permeability of the bacterial cell membrane of Gram-negative bacteria and promote the leakage of Ca^2+^, Na^+^ and other cations. This study revealed that MPX can produce bactericidal effects on MDR strains, destroy the structure of bacterial cell membranes, increase the permeability of bacterial cell membranes, change the membrane potential of bacteria, cause deformation and rupture of bacteria, and cause leakage of cell contents, thereby producing a sterilization effect.

Biofilms are microbial aggregates that adhere to surfaces and grow in an organized manner. Unlike planktonic cells, biofilm-resident cells have better adhesion to animal tissues and are more resistant to antibacterial drugs. Biofilms are an important cause of antibiotic resistance, which produces tremendous challenges for clinical treatment. Therefore, the development of microbial biofilm-attenuating agents provides an effective way to prevent and treat such infections. According to reports, the antimicrobial peptide 1,4-naphthoquinone can inhibit the biofilm formation of the Gram-positive bacterium *S. aureus* through the accumulation of cellular ROS (27). The antimicrobial peptide AMPNT-6 can effectively prevent attachment and biofilm formation by the Gram-negative bacterium *Shewanella putrefaciens* (28). The antibacterial peptide Ctn [15–34] showed antibiofilm activity against fungi (29). The synthetic peptide SHABP can reduce the formation of oral plaque biofilms, which provides a reference for the development of oral antibiofilm preparations (30). Therefore, AMPs can be used as a new way to treat biofilm-related infections and AMPs can provide references for the development of other antibiofilm formulations. This study revealed that MPX could inhibit bacterial biofilm formation. MPX (0.5×MIC) significantly reduced *S. aureus* biofilm formation and has the potential to be developed as an antibacterial drug.

The skin barrier system can prevent the entry of harmful environmental substances (including invasive microorganisms) and is very important in maintaining physical, chemical and immune barriers. When the normal skin barrier is destroyed, such as by scratches and burns, pathogenic bacteria can colonize the wound and cause infection. Wound infection is an important factor affecting wound healing. However, with the increase in the incidence of drug-resistant bacterial infections, traditional antibiotic treatment has become increasingly challenging (23). To effectively avoid the drawbacks of traditional antibiotic treatment, we should pay attention to the research and development of new formulations. TA-Fe-based nanohydrogels have been reported to effectively heal wound infections and promote wound healing (31). A modular adaptive electrotherapy delivery system (MAEDS) can significantly accelerate wound healing and significantly reduce bacterial infection, providing a new therapeutic avenue for wound healing (32). Wound therapy based on AMPs is also a research hotspot. The antimicrobial peptide CAMP has been reported to significantly reduce the colonization of skin wounds by *Pseudomonas aeruginosa*, promote wound healing, and prevent bacterial spread in the liver (33). Antimicrobial peptide-functionalized mesoporous hydrogels can reduce the colonization of drug-resistant *S. aureus* in a mouse model of skin wounds (34). The artificially modified antimicrobial peptide Pse-T2 can promote wound healing, reduce colonization, and reduce inflammation (26). This study revealed that the MPX-based ointment can reduce wound colonization and promote wound healing. It can not only reduce the inflammation of skin wound tissue but also protect the lungs from bacterial invasion, providing a basis for the study of wound dressings.

In summary, we systematically evaluated the antibacterial activity of the antimicrobial peptide MPX on the basis of *S. aureus* and clarified the antibacterial mechanism of MPX. It was found that MPX can inhibit biofilm formation, destroy cell membrane integrity, change the cell membrane potential, and cause content leakage, thereby mediating bacterial death. Furthermore, MPX ointment can reduce inflammation by reducing wounds colonization, thereby promoting healing of wounds caused by *S. aureus* infection. These findings show that MPX has significant potential to treat bacterial wound infections and provides a basis for the research and development of new antibacterial agents.

## Data Availability Statement

The original contributions presented in the study are included in the article/Supplementary Material, further inquiries can be directed to the corresponding author/s.

## Ethics Statement

The research was approved by the Animal Center of Henan Institute of Science and Technology.

## Author Contributions

LW, XZhan, and JH conceived the idea for this study. SL, XX, SZ, YWa, and HZ were involved in the conception and design of the study. XZhao, YWu, XW, and HF performed the experiments and analyzed and interpreted the data. GZ, YB, YX, SC, and JJ were involved in drafting and revising the manuscript. XZhan also co-devised the idea for the study and provided funding support. All authors contributed to the article and approved the submitted version.

## Funding

This work was supported by the National Natural Science Foundation of China (32172862), the Young Talent Lifting Project in Henan Province (2020HYTP041), the Key Scientific Research Projects of Colleges and Universities inHenan Province (21A230004), the National Key Research and Development Program of China (2019YFC605700), the Open Project of the State Key Laboratory of Marine Resources Utilization in the South China Sea (Hainan University, MRUKF2021004), the Youth Backbone Teacher Project of Colleges and Universities of Henan Province (2020GGJS162), and the Innovative Research Team (in Science and Technology) in University of Henan Province (20IRTSTHN025).

## Conflict of Interest

The authors declare that the research was conducted in the absence of any commercial or financial relationships that could be construed as a potential conflict of interest.

## Publisher's Note

All claims expressed in this article are solely those of the authors and do not necessarily represent those of their affiliated organizations, or those of the publisher, the editors and the reviewers. Any product that may be evaluated in this article, or claim that may be made by its manufacturer, is not guaranteed or endorsed by the publisher.

## Supplementary Material

Supplementary Figure 1 Proteinase K treatment on the antibacterial activity of MPX. Error bars indicate the mean ± SEM, *n* = 3. Statistical significance was defined as ^****^*P* < 0.0001.

Supplementary Figure 2 Trypsin treatment on antibacterial activity of MPX. Error bars indicate the mean ± SEM, *n* = 3. Statistical significance was defined as ^****^*P* < 0.0001; ns *P* > 0.05.
