# Supplementary figures and images for: The Antimicrobial Peptide MPX Can Kill Staphylococcus aureus, Reduce Biofilm Formation, and Effectively Treat Bacterial Skin Infections in Mice
Source: Front Vet Sci. 2022 Mar 29;9:819921. doi: 10.3389/fvets.2022.819921 (PMC9002018; doi:10.3389/fvets.2022.819921)

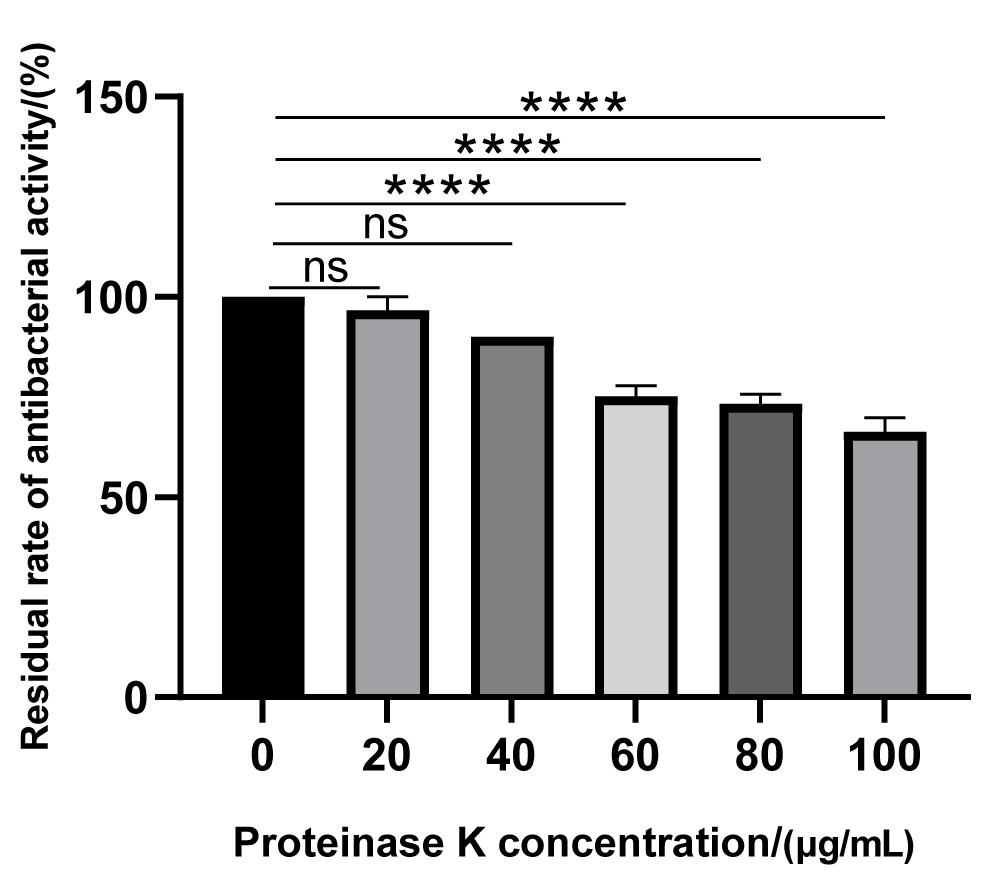

Supplement: Supplementary Figure 1 — Proteinase K treatment on the antibacterial activity of MPX. Error bars indicate the mean ± SEM, n = 3. Statistical significance was defined as ****P < 0.0001. [file Image_1.jpeg]

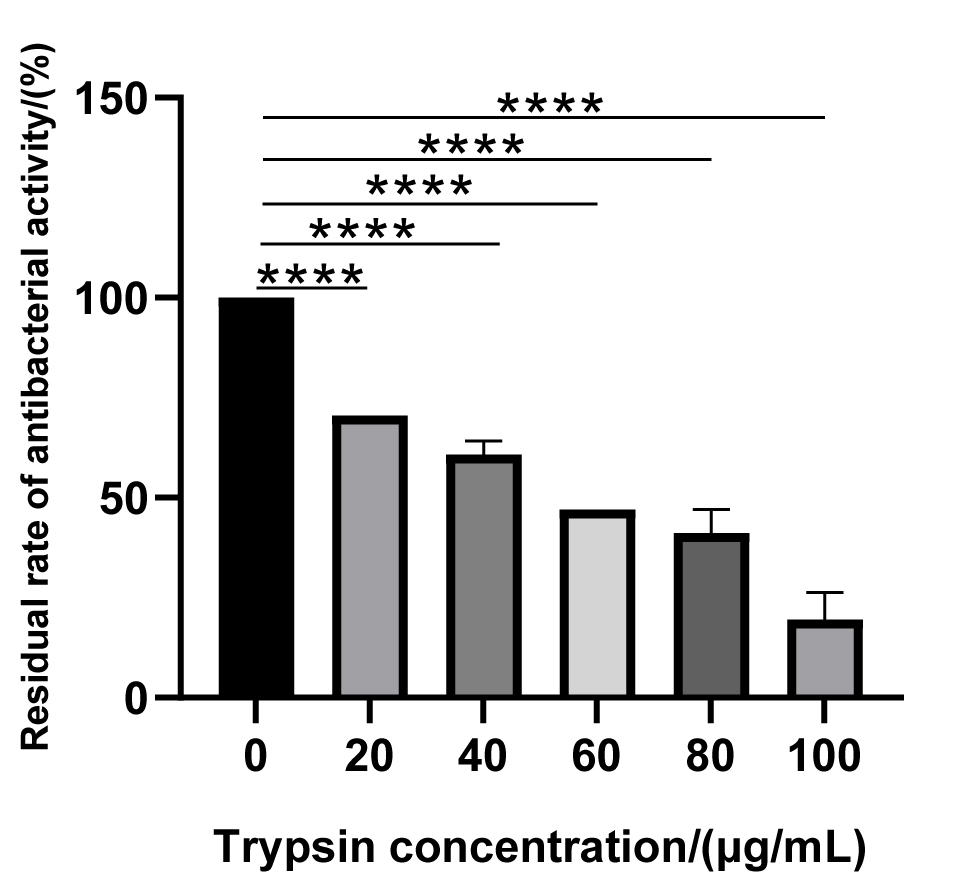

Supplement: Supplementary Figure 2 — Trypsin treatment on antibacterial activity of MPX. Error bars indicate the mean ± SEM, n = 3. Statistical significance was defined as ****P < 0.0001; ns P > 0.05. [file Image_2.jpeg]
